# Identification of Two Novel Mutations in *COG5* Causing Congenital Disorder of Glycosylation

**DOI:** 10.3389/fgene.2020.00168

**Published:** 2020-02-27

**Authors:** Xi Wang, Lin Han, Xiao-Yan Wang, Jian-Hong Wang, Xiao-Meng Li, Chun-Hua Jin, Lin Wang

**Affiliations:** ^1^Department of Preventive Health Care, Children’s Hospital, Capital Institute of Pediatrics, Beijing, China; ^2^Running Gene Inc., Beijing, China.

**Keywords:** *COG5* gene, congenital disorder of glycosylation, psychomotor delay, ataxia, visual abnormalities, genetic sequencing

## Abstract

**Objective:**

This study reports a Chinese patient with a Congenital Disorder of Glycosylation (CDG) caused by compound-heterozygous mutations in the Conserved Oligomeric Golgi 5 (*COG5*) gene and thereby offers concrete evidence for early diagnosis.

**Methods:**

The clinical manifestations, the results of laboratory examinations and genetic analysis of a 4-year-old Chinese girl with CDG are reported. We also reviewed previous CDG cases that involved *COG5* mutations by comparing the phenotypes and genotypes in different cases.

**Results:**

The patient was admitted to our hospital due to ataxia and psychomotor delay. The major clinical manifestations were postural instability, difficulty in walking, psychomotor delay, hypohidrosis, hyperkeratosis of the skin, and ulnar deviation of the right-hand fingers. Biochemical analyses revealed coagulation defect and liver lesions. Vision tests showed choroidopathy and macular hypoplasia. Whole-exome sequencing identified the hitherto unreported compound-heterozygous *COG5* mutations, c.1290C > A (p.Y430X) and c.2077A > C (p.T693P). Mutation p.Y430X is nonsense, leading to a truncated protein. Mutation p.T693P is located at a highly conserved region, and thus the polar-to-non-polar substitution presumably affects the structure and function of COG5. According to the Human Genome Mutation Database Professional, there have been totally 13 CDG cases caused by 13 *COG5* mutations. They are mainly characterized by psychomotor delay, hypotonia, ataxia, microcephaly, and hearing and visual abnormalities.

**Conclusion:**

The clinical manifestations of the patient are mild but consistent with the clinical characteristics of the published COG5-CDG cases. The results of this study extend the spectrum of clinical and genetic findings in COG5-CDG.

## Background

Congenital Disorders of Glycosylation (CDGs) represent a group of metabolic disorders resulting from defects in the glycosylation of proteins. Most of CDGs are autosomal recessive, but autosomal dominant and X-linked ones have also been reported. The classification of CDGs depend on the defective enzyme and its function. More than 130 types of CDGs characterized to date involve enzymes participating in various steps along glycosylation pathways (Chang et al., 2018). Conserved Oligomeric Golgi 5 (COG5) is a subunit of the COG complex which functions in trafficking and glycosylation as well as the maintenance of structure of Golgi apparatus (Blackburn et al., 2019). The deficiency of COG5 leads to COG5-CDG, which is a rare type and usually caused by mutations in the *COG5* gene. Its clinical manifestations may include neurological problems among other abnormalities and the pathogenesis is unclear. Only 7 of 13 cases have provided clinical information in detail to date. Here, we report a 4-year-old Chinese patient carrying two novel compound-heterozygous mutations in *COG5*, whereby we expand the mutation database.

## Case Presentation

The patient was admitted to the department of preventive health care in the Children’s Hospital of Capital Institute of Pediatrics due to ataxia and psychomotor delay. She was the first child of her parents, and her mother had no pregnancy before. The birth occurred 38 weeks post-gestation. The height and weight of the patient at birth were 49 cm and 3000 g, respectively. She presented with neonatal jaundice but it completely subsided 35 days after birth. In addition, the patient had recurrent upper respiratory infections approximately five times a year.

**FIGURE 1:**
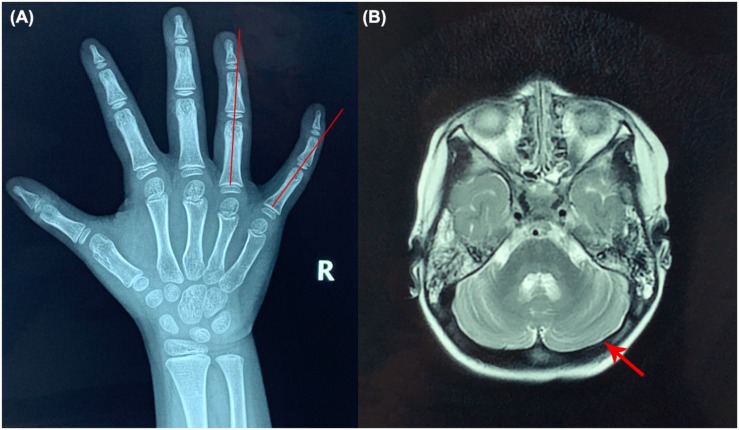
X-ray of the right hand and brain MRI of the patient. **(A)** Ulnar deviation of the right-hand ring finger and little finger (red lines). **(B)** Brain MRI results (red arrow shows the cerebella).

Upon admission, her height, weight and BMI were 104.7 cm (35th percentile), 18.1 kg (76th percentile), and 17.42 kg/m^2^ (91st percentile), respectively. Her head circumference was 48 cm. She had convergent strabismus and could not respond to any communication attempt. Hypohidrosis, hyperkeratosis of the skin at the dorsa of the hands and ulnar deviation of the right-hand ring finger and litter finger (Figure 1A) were identified, but no hypotonia was observed in the examination. We could not perform an ataxia text due to the psychomotor delay of the patient.

Biochemical examinations showed the elevated ALT and AST levels (ALT 48.3 U/L and AST 55.3 U/L, respectively; reference values [r.v.] are 0–40.0 U/L), but liver ultrasound revealed nothing abnormal. The activated partial thromboplastin time (APTT) was 40 s ([r.v.] 24–37 s). The lymphocyte count and immunoglobin level of the patient were normal. No abnormalities were found in brain MRI (Figure 1B).

The Gesell Development Scale (Conoley and Impera, 1995) results revealed that the developmental quotient of the patient was 43 and her intelligence age was 23.4 months. Her Clancy Autism Behavior Scale (CABS) (Zachary Warren and Howell Dohrmann, 2013) and Infants-Junior High School Students’ Social Life Abilities Scale (S-M scale) (Zhang et al., 1995) were 9 and 10, respectively.

## Methods

To confirm the diagnosis, genetic sequencing was performed with 5-ml peripheral blood samples from the patient and her parents. The samples were collected in our hospital and then sent to Running Gene Inc. (Beijing, China) for sequencing. DNA samples were extracted and purified by DNA Isolation Kit (AU1802, Bioteke) and Agencourt AMPure XP kit (Beckman Coulter, Inc., United States), respectively. Hybridization was performed using the IDTxGen Exome Research Panel v1.0 (Integrated DNA Technologies, Inc., United States). Targeted DNA samples were sequenced by the Illumina Novaseq 6000 system (Illumina, United States), and quality control was applied to the raw data (stored in FASTQ format) by Illumina Sequence Control Software (SCS). High-quality data were aligned to the human reference genome sequence hg19^1^ using BWA tool^2^. Consensus single nucleotide variants (SNV) and insertion/deletions (INDEL) were filtered via GATK^3^. All SNV and INDEL information was annotated by the software ANNOVAR^4^. The pathogenicity of candidate mutations was analyzed based on the American College Medical Genetics and Genomics (ACMG) guidelines (Richards et al., 2015). Sanger sequencing was used to validate the family segregation of mutations.

The expression level of COG5 proteins has been demonstrated by western blot. Leukocytes of the patient and control were extracted from their peripheral blood samples by human peripheral blood leukocyte separation kit (P8670, Solarbio, China). Extracted cells were mixed with RIPA buffer and broken by ultrasonication. After centrifugation, the concentrations of supernatants were measured by BCA protein assay kit (CW0014S, Cowin Bio, China). Proteins were separated by SDS-PAGE and immunoblotted. Signals were detected by ECL kit (P0018FM, Beyotime, China), and the quantification was performed using ImageJ2 (Rueden et al., 2017). Anti-COG5 (ab229830), anti-GAPDH (ab8245), goat anti-rabbit IgG H&L (HRP) (ab205718), and goat anti-mouse IgG H&L (HRP) (ab205719) were purchased from Abcam, United Kingdom. All antibodies were diluted as 1:1000.

## Results

### Laboratory Examinations

**FIGURE 2:**
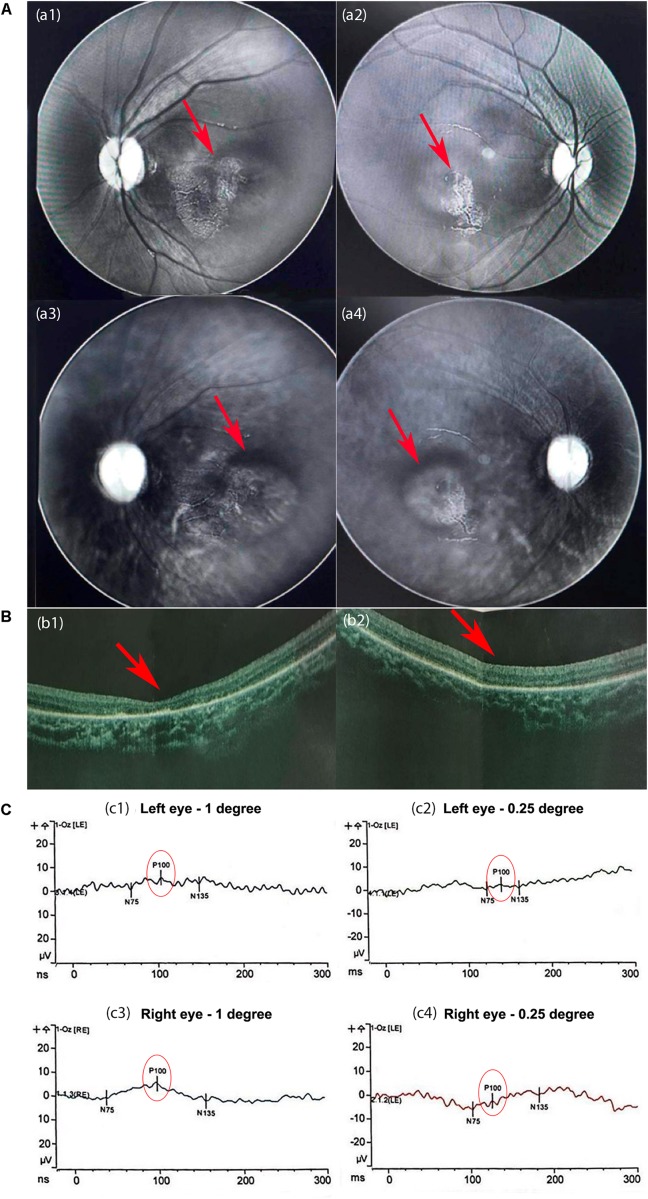
The results of the eye examination. **(A)** Multispectral retinal image analysis. Abnormal fluorescence on the left **(a1)** and right **(a2)** retinas. Mass shadows in the macular region of the choroid in the left **(a3)** and right **(a4)** eyes. **(B)** Optical coherence tomography (OCT) images. The fovea centralis was absent from the center of the macula lutea of the left **(b1)** and right **(b2)** retinas (red arrows). The retinal pigment layers were intact. **(C)** The results of the pattern visual evoked potential (PVEP). At 1-degree checks, the latencies of P100 were normal but the amplitudes of P100 were moderately reduced in the left eye **(c1)** and slightly in the right eye **(c3)**. **(c2**,**c4)** At 0.25-degree checks, the latencies of P100 were significantly longer, and the amplitudes were severely reduced in both eyes.

An extension of APTT revealed defective coagulation. Lymphocyte count and quantitation of immunoglobin level indicated a normal immune system. Acoustic impedance tests showed normal results. Multispectral retinal image analysis of binoculus revealed abnormal fluorescence on the retina and mass shadows in the macular region of the choroid (Figure 2A). Optical Coherence Tomography (OCT) detected no fovea centralis in the center of the macula lutea of the retina (Figure 2B). Pattern visual evoked potential (PVEP) test revealed that the latencies of P100 waves at 1-degree checks were roughly normal but the P100 amplitudes were moderately reduced in both eyes; the latencies at 0.25-degree checks were significantly longer, and the amplitudes were severely reduced in both eyes, suggesting choroiditis in the retina and hypoplasia in the macula lutea (Figure 2C).

### Genetic Sequencing

**FIGURE 3:**
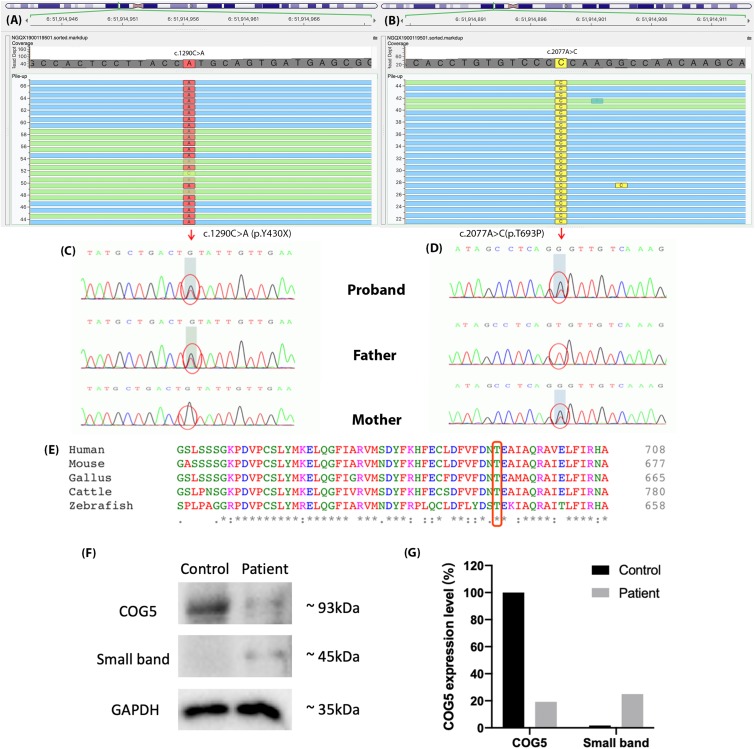
The results of genetic sequencing. Two mutations were identified in *COG5*, **(A)** c.1290C > A (p.Y430X) and **(B)** c.2077A > C (p.T693P). **(C**,**D)** The father of the proband carried mutation c.1290C > A (p.Y430X), and her mother carried c.2077A > C (p.T693P). The compound-heterozygous mutations in the proband were inherited from her parents. **(E)** Thr693 is highly conserved across species and located in a highly conserved region. **(F)** Expression levels of COG5 (93 kDa), small band (around 45 kDa) and GAPDH (Reference, 35 kDa) were shown by western blotting. **(G)** Quantification of the protein levels. The expression level of COG5 proteins was lower in the patient compared with control. A unknown small band appeared in the patient’s sample but control.

We identified two novel compound-heterozygous mutations in the *COG5* gene (NM_006348.3). Sanger sequencing of the DNA samples from the parents of the proband validated this result (Figures 3A–D). She inherited the nonsense mutation, c.1290C > A (p.Y430X), from her father and the missense mutation, c.2077A > C (p.T693P), from her mother. According to the Human Genome Mutation Database Professional version (HGMD Pro, 20190711 updated) (Richards et al., 2015), neither of these mutations has been reported before.

### Western Blotting

The expression levels of COG5 proteins and GAPDH were shown by western blotting (Figure 3F). The protein levels were quantified and normalized (Figure 3G). It illustrated that the expression level of COG5 proteins in the patient was lower compared with a healthy control. Meanwhile, a band with smaller size (around 45 kDa) showed a stronger signal in the patient’s sample but control.

## Discussion

Congenital Disorders of Glycosylation are disorders in the glycosylation of proteins, classified as CDG I and II. CDG I encompasses defects in the biosynthesis of lipid oligosaccharide chains or in the transformation of sugar chains to protein precursors, whereas CDG II involves defects in subsequent trimming of the oligosaccharide chains and glycosylation of terminal sugars (Wu et al., 2004; Kim et al., 2017). COG5-CDG is a subtype of CDG II, affecting modification pathways of N-linked oligosaccharide (Sparks and Krasnewich, 2017). The COG complex, as a membrane protein, plays an essential role in the maintenance of the Golgi structure, glycosylation of proteins and retrograde transport of vesicles in the Golgi apparatus (Zeevaert et al., 2008; Jaeken, 2011; Blackburn et al., 2019). The COG complex is composed of 8 subunits, including lobe A (from subunit COG1 to COG4) and lobe B (from subunit COG5 to COG8) (Rymen et al., 2012). Lobe A and B bind each other via interactions between subunits COG1 and COG8. In humans, mutations in gene *COG1*, *COG4*, *COG5*, *COG6*, *COG7*, and *COG8* have been reported to be associated with CDG (Wu et al., 2004; Foulquier et al., 2006; Foulquier et al., 2007; Reynders et al., 2009; Rymen et al., 2015; Kim et al., 2017).

In this study, the patient carried two novel mutations in *COG5*, c.1290C > A (p.Y430X) and c.2077A > C (p.T693P). The nonsense mutation p.Y430X presumably leads to the loss-of-function of gene *COG5* (PVS1) and is extremely infrequent in GnomAD, ExAC and 1000 Genomes (PM2) (Genomes Project et al., 2015; Lek et al., 2016; Karczewski et al., 2019). Several mutations downstream of p.Y430X have previously been reported as disease-causing, including p.V594F, p.I640T, p.P775L, and p.E840X. Since p.Y430X is a nonsense mutation, a truncated COG5 protein that lacks residues V594, I640, P775, and E840, forms and likely causes the disease. Therefore, p.Y430X is classified as “likely pathogenic,” according to the ACMG guidelines. Moreover, the missense p.T693P is absent from 1k Genome database and extremely infrequent in ExAC and GnomAD databases (PM2). Additionally, it was located in *trans* with p.Y430X in the patient (PM3). Thus, p.T693P is considered as a variant of uncertain significance (VUS). However, this missense mutation was in a highly conserved region (Figure 3E), indicating the importance of the mutated residue. Whereas threonine is an uncharged polar amino acid, but Proline carries a charged residue. Thus, the alteration of the residue characteristics might impair the structure and functions of the final product. We considered both p.Y430X and p.T693P are disease-causing mutations.

As shown in Figures 3F,G, the expression levels of COG5 proteins were declined in the patient, who carried two mutations. Based on the location of the nonsense mutation (p.Y430X) and the size of COG5 (93kDa) and the small band (45kDa), we considered it as the aberrant COG5 protein. Our reasonable speculation is supported by previous research which also identified a small band around 40kDa in the patient with p.M403IfsX3 (Kim et al., 2017). The amino acid sequence of the smaller protein could be determined to find out whether or not it is the truncated COG5 in the further research. Although we cannot prove it is the aberrant COG5 for now, the results from western blot indeed indicate the deficiency of normal COG5 in our patient.

According to the HGMD pro, there have been totally 13 CDG cases caused by 13 COG5 mutations. Only 8 COG5-CDG cases carrying 10 COG5 mutations have been reported in detail to date, including the one characterized in this study (Table 1) (Paesold-Burda et al., 2009; Fung et al., 2012; Rymen et al., 2012). The patients in all the cases had intellectual disability (ID) and developmental delay, and some patients suffered from unstable gait and delayed speech functions, similar to the manifestations observed in this study. Most of the patients had hypotonia (7/8). Four of patients also had abnormal MRI results, including myelination delay, cerebellar atrophy and reduced white matter. However, these symptoms are absent in the case reported here, and the brain MRI showed a generally normal result. These observations are consistent with the statement that hypotonia might be correlated with brain lesion (Paesold-Burda et al., 2009; Fung et al., 2012; Rymen et al., 2012).

**TABLE 1:** Genetic and clinical features of COG5-CDG patients whose data are available.

Case	Ref	M/F	Age of onset	Race	Mutation	Allele frequency in GnomAD	ID/development delay 8/8	Hypotonia 7/8	Brain MRI 4/8	Microcephaly 6/8	Vision abnormalities 5/8	Neurogenic bladder 4/8	Ataxia 3/8	Liver lesion 3/8	Facial dysmorphism 2/8	Others
**1**	Present case	F	4 years	Chinese	c.2077A > C, p.T693P; c.1290C > A, p.Y430X	0.000004; 0.000012	+/+	–	–	–	Choroidopathy; hypoplasia of macula; convergent strabismus	–	+	Increased ALT and AST levels	–	Hypohidrosis, hyperkeratosis; deviation of finger; coagulation defect
**2**	Paesold-Burda et al. (2009)	F	8 years	Iraqi	c.1669-15A > G	0	+/+	+	Diffuse atrophy of cerebellum and brain stem	–	Ocular motor apraxia	–	+	–	–	–
**3**	Fung et al. (2012) and Rymen et al. (2012) P2	F	1 month	Chinese	c.556_560 delAGTAAinsCT; c.1919T > C, p.I640T; c.95T > G, p.M32R	0; 0.00156037; 0.0000323206	+/+	+	Delayed myelination	+	–	–	–	Liver cirrhosis; mild hepato- splenomegaly	–	Mild thrombocytopenia; persistent mild hyperlactacidemia; portal hypertension; fixed flexion contractures of all fingers
**4**	Rymen et al. (2012) P1.1	F	1 year	Moroccan	c.2518G > T, p.E840X	0	+/+	+	Global decrease of white matter; enlarged lateral ventricles	+	–	–	–	–	Posteriorly rotated, low set ears, a prominent nose and low hair line	–
**5**	Rymen et al. (2012) P1.2	F	N/A	Moroccan	c.2518G > T, p.E840X	0	+/+	+	N/A	+	–	+	+	–	–	Slight dysmorphism; autistic behavior
**6**	Rymen et al. (2012) P1.3	F	8 months	Moroccan	NA	–	+/+	+	–	+	Strabismus	+	–	–	–	Autistic behavior
**7**	Rymen et al. (2012) P3	M	3 months	Italian	c.189delG, p.C64Vfs*6; c.2338_2340 dupATT, p.I780dup	0.0000322872; 0	+/+	+	Severe supra- and subtentorial brain atrophy	+	Strabismus; cortical blindness	+	N/A	hepatomegaly	–	Sensorineural deafness; recurrent urinary tract infection; spastic quadriplegia; scoliosis
**8**	Rymen et al. (2012) P4	M	At birth	Belgian	c.1780G > T, p.V594F	0	+/+	+	–	+	Cortical blindness	+	N/A	–	Low set, posteriorly rotated ears, a prominent nose with a broad root and retrognathia	Sensorineural deafness; hypohidrosis, epilepsy; micropenis with cryptorchidism; campodactyly and clinodactyly; flexion contractures of knees and elbows

*NA = not available. All mutations are stated as in NM_006348.*

Other clinical manifestations are microcephaly (6/8), visual abnormalities (strabismus, cortical blindness, etc.) (5/8), neurogenic bladder (4/8), ataxia (3/8), liver lesion (3/8), and facial dysmorphism (2/8). Microcephaly was absent in this case. Since hearing and visual abnormalities, such as sensorineural deafness, strabismus and cortical blindness have been reported in COG5-CDG patients before (Rymen et al., 2012), we assessed the hearing and visual capabilities of the patient. Convergent strabismus, choroidopathy, and macular hypoplasia were diagnosed. Since there have hitherto been 5/8 patients diagnosed with visual abnormalities, we herein recommended a regular ophthalmic examination upon admission of COG5-CDG patients and follow-ups every year. The examination is widely used in other CDG patients. The major ophthalmological manifestations of PMM2-CDG (phosphomannomutase2-CDG, the commonest type) are also strabismus, nystagmus, pigmentary retinopathy, reduced visual acuity, and myopia (Altassan et al., 2019). All these symptoms may share a similar unknown pathogenic mechanism with COG5-CDG. Although there is no targeted therapy for these visual abnormalities, eyeglasses or eye surgery can be applied to correct visual acuity.

Moreover, ALT and AST levels were elevated in the patient presented here, suggesting a metabolic disorder in the liver. The liver is the major location of glycosylation, and the majority of glycosylated serum proteins are generated by it. The dysfunction of glycosylation can impair the structure and functions of the liver, elevating the mRNA and protein levels of ALT and AST and subsequently exacerbating the liver lesion (Marques-da-Silva et al., 2017). Thus, the impaired liver function is commonly observed in CDG patients. Although there is no radical cure, associated complications, such as ascites, liver failure, bleeding tendency and hepatopulmonary syndrome can be treated. Therefore, assessment of liver function and imaging of the abdomen are suggested to be regularly performed for prevention and early diagnosis.

In addition, glycosylation plays critical roles in the maturation of immune cells and the recognition between antibodies and antigens, modulating innate and adaptive immunities (Rudd et al., 2001). Recurrent infections have been found in COG6-CDG patients with simultaneous T-/B-cell and neutrophil dysfunctions (Huybrechts et al., 2012). Recurrent infections were noted in the present case (upper respiratory tract) and case 7 (urinary tract) as well. However, the immune system of the patient reported here had no defect. Furthermore, no immune deficiency has been reported in any COG5-CDG patients.

The patient described in this study is the only COG5-CDG patient reported to have a coagulation defect. Previously, coagulation disturbance has been presented in MPI-CDG, ALG12-CDG, ALG2-CDG, ALG1-CDG, and B4GALT1-CDG, and it has been one of the most commonly reported clinical symptoms in CDG patients. Both bleeding and thrombosis risks are high in CDG patients due to the altered levels of serum coagulation factors, such as factors IX and XI, Protein C, Protein S, and antithrombin III. However, the internal mechanism is still elusive, and there is no targeted treatment. Thus, it is strongly recommended that the coagulation statues of CDG patients are regularly monitored (Krasnewich et al., 2007).

In our study, the child had mild symptoms, mild ID and ataxia without hypotonia, microcephaly and facial dysmorphism. The clinical characteristics among COG5-CDG patients revealed a massive difference from mild ID and normal facies to severe ID, hypotonia, microcephaly, facial dysmorphism and multiple organ involvement. The declined protein levels of COG5 and COG7 has been identified via western blot in COG5-CDG patients with different clinical features, and the patients with severe symptoms had clinical manifestations overlapped with COG7-CDG patients (Rymen et al., 2012). Since protein COG5 and COG7 are responsible for the formation of the stable complex lobe B, the interactions of protein levels might affect the severity of patients’ phenotypes.

Currently, there is no specific therapy for COG5-CDG patients. However, following symptomatic treatments can be performed: surgical treatment of patients who have finger contracture affecting finger function, gastrostomy or nasal feeding of children who suffer from feeding difficulties, and bladder ostomy on patients who have neurological bladder. No death has been reported in any of the cases we reviewed. In 4/8 cases, the intelligence level gradually improved, and the myelination delay was improved in the present case (Jaeken, 2011; Fung et al., 2012; Rymen et al., 2012). The follow-up visit of the patient reported here revealed that her intelligence development progressed after a 2-month intervention.

To date, 8 CDG cases with detailed clinical data have been reported to be associated with *COG5* mutations. Here, we diagnosed a 4-year-old Chinese girl as COG5-CDG based on clinical manifestations and whole-exome sequencing. The clinical manifestations of this patient were mild ID, ataxia, convergent strabismus, choroidopathy, macular hypoplasia, coagulation defect, and elevated AST and ALT levels. All these manifestations were mild but consistent with the clinical characteristics of described COG5-CDG cases. Genetic sequencing can offer concrete evidence for early assessment, diagnosis and treatment for CDG patients with similar symptoms.

## Data Availability Statement

The raw data supporting the conclusions of this article will be made available by the authors, without undue reservation, to any qualified researcher.

## Ethics Statement

The studies involving human participants were reviewed and approved by the Research Ethics Committee of Children’s Hospital of Capital Institute of Pediatrics. Written informed consent to participate in this study was provided by the participants’ legal guardian/next of kin. Written informed consent was obtained from the individual(s), and minor(s)’ legal guardian/next of kin, for the publication of any potentially identifiable images or data included in this article.

## Author Contributions

XW and LW designed the study and cared the patient. XW and LH wrote the manuscript. XW, LH, X-ML, and C-HJ collected and analyzed the clinical data and published cases. LH contributed to the experiment. XW, LH, and LW reviewed the manuscript. LW, X-YW, and J-HW supervised this study. All authors read and approved the submitted version.

## Conflict of Interest

LH is employed by Running Gene Inc., Beijing, China. The remaining authors declare that the research was conducted in the absence of any other commercial or financial relationships that could be construed as a potential conflict of interest.

## Footnotes

1 http://genome.ucsc.edu/

2 http://bio-bwa.sourceforge.net/

3 https://software.broadinstitute.org/gatk/

4 http://annovar.openbioinformatics.org/en/latest/
